# Effect of School Bullying on Students’ Peer Cooperation: A Moderated Mediation Model

**DOI:** 10.3390/children11010011

**Published:** 2023-12-21

**Authors:** Yu-Jiao Wang, I-Hua Chen

**Affiliations:** 1 School of Education Science, Liupanshui Normal University, Liupanshui 553004, China; 2 Chinese Academy of Education Big Data, Qufu Normal University, Qufu 273100, China; chenih0807@qfnu.edu.cn

**Keywords:** school bullying, peer cooperation, school belonging, teacher support, parents’ support

## Abstract

Background: Studies show that cooperative environments enhance student performance. However, school bullying can significantly undermine peer cooperation. There is limited research on how school bullying impacts peer cooperation and the mechanisms involved. Methods: Using data from 15-year-old middle school students in four Chinese provinces and cities, as part of the 2018 Program for International Student Assessment (PISA), this study employs a moderated mediation model. It examines the negative effects of school bullying on peer cooperation, the mediating role of school belonging, and the moderating effects of teacher support and parents’ support. Results: School bullying negatively impacts peer cooperation. School belonging partially mediates this relationship. Teacher support moderates the effect of school bullying on school belonging, which in turn affects peer cooperation. Parents’ support moderates the direct impact of school bullying on peer cooperation. Conclusion: School bullying reduces peer cooperation by diminishing students’ sense of belonging in school. This effect is lessened with increased support from teachers and parents. The findings suggest that while social support is beneficial, it must be balanced and not excessive.

## 1. Introduction

In the 21st century, the ability to cooperate with others in solving problems has become an indispensable core social skill for the new generation, essential for adapting to the needs of the new era [1]. The benefits of cooperative behaviors have been widely proven across various social environments, including communities, hospitals, and companies [2,3,4]. For example, during the COVID-19 crisis, greater cooperation among countries and groups worldwide reduced the health, social life, and economic harm impacts of the epidemic by increasing compliance with social distancing advice [5].

In education, empirical evidence suggests that students in cooperative academic environments not only excel academically but also report enhanced relationships with peers and stronger school attachment, compared to those in competitive contexts [6,7]. Trust and collaboration among students, teachers, parents, and principals particularly benefit disadvantaged students [8,9,10]. Thus, schools should focus on enhancing students’ ability to cooperate and actively create environments conducive to effective cooperation. Cooperative behavior is diverse and complex. According to social cognitive theory, individual, environmental, and behavioral factors are interdependent yet each exerts a causal influence [11,12,13]. While individual factors often drive cooperative behavior, environmental factors provide the conditions for its maintenance [14]. Previous studies on cooperative behavior have mainly concerned individual factors such as personality traits, social value orientation, motivation, and environmental factors such as reward and punishment, individual–collectivism cultural backgrounds, group identity, etc. [14]. Although existing studies have emphasized the importance of environmental factors in student cooperation, the impact of peer relationships, especially the destructive role of school bullying, has been less explored. Cooperation is a socialized behavior, and good peer relationships contribute to cooperative behaviors, whereas undesirable relationships, particularly those affected by school bullying, can cause great damage to good cooperation [15]. However, there is no discussion on the mechanism of the impact of campus bullying on peer cooperation.

School bullying, defined as repeated and deliberate aggressive behavior by one or more students towards a peer [16], can include physical, verbal, or other forms of harm [17,18]. The power imbalance in school bullying often makes it difficult for victims to resist [19,20,21]. Such bullying significantly disrupts peer relationships, leading to a negative attitude towards cooperation among victims. This study aims to explore the relationship and mechanism of school bullying on peer cooperation, to enrich the theoretical understanding of these dynamics, and to provide new strategies for preventing campus bullying and enhancing cooperative atmospheres in schools. 

### 1.1. School Bullying and Peer Cooperation

School bullying is an important issue of worldwide concern, and being bullied can cause serious consequences to students’ health. Research identifies bullying as a significant risk factor for adolescent mental and physical health, both short and long term [22]. Victims of bullying are prone to depression, anxiety, low self-esteem, loneliness, and sadness [23,24,25], and often exhibit disengagement from school, impaired social relationships, and diminished academic performance [26,27,28]. According to the frustration–aggression theory [29], both children and adults are prone to anger and other emotions related to aggression after being frustrated, and their aggressive behavior will increase or be further strengthened, and the peer relationship is then violated. Some studies have explored the effect of bullying on peer relationships; for example, a survey of 827 primary and middle school students identified a negative correlation between school bullying and peer acceptance [30]. Also, there are some discussions on peer relationships focused on peer support [31], peer fear and low self-esteem [32], peer acceptance and rejection [33], trouble with peer friendship [34], peer conflict [35], undesired companions relationship [36], and other aspects. According to the analysis of previous research results, students who suffer from school bullying will feel that they receive serious injury by peer groups, a lack of interest in school ensues, and social relations become impaired [26,27], which may lead to a negative perception of the cooperative relationship between classmates. This leads to our first hypothesis H1:

**H1.**  *School bullying negatively impacts peer cooperation*.

### 1.2. The Mediating Role of School Belonging

School is a crucial setting for children’s social interaction, trust-building with teachers, and attachment formation [37]. According to Maslow’s hierarchy theory of needs, the need of belonging and love is individual’s basic need, influencing students’ psychological development [38,39]. However, if they are bullied at school, it is very difficult for them to form the school belonging [40]. Studies have shown that school bullying impairs the formation of school belonging [41,42], and increases the proportion of truancy as well as academic and test anxiety [42,43]. There is also evidence of a negative mutual relationship between school bullying and school belonging [44]. Longitudinal studies have found that positive changes in school belonging can predict a reduction in school bullying behaviors [45]. A sense of school belonging also mediates the relationship between peer support and school bullying [31,36]. 

A student’s sense of belonging to school refers to the degree to which a student feels accepted, respected, or supported by teachers and classmates in the school [46,47], indicating that they see themselves as part of the school groups. According to the social identity theory or group identity theory, individuals identify with their own groups through social classification, and produce in-group and out-group preferences [48]. Eaton, Eswaran, and Oxoby (2011) found that individuals’ intrinsic tendency to classify “insiders” and “outsiders” differently, namely, their personal identity, affects cooperation [49]. Contrary evidence also suggests that group heterogeneity, such as group members belonging to different races or religions, is detrimental to cooperation [50]. Previous research revealed that people with a high sense of belonging will also have more cooperative behaviors [51]. This argument is consistent with the results of a recent study, which shows that cooperation can increase a sense of inclusion, thereby satisfying the need for sense of belonging [52]. In summary, students who are bullied at school have a reduced sense of school belonging, which in turn will reduce their intention to cooperate. So, this study speculates the second hypothesis, H2.

**H2.**  *School belonging mediates the impact of school bullying on peer cooperation*.

### 1.3. The Moderating Role of Teacher Support and Parents’ Support

Mills’ significant others theory posits that parents, teachers, and peers are important in the socialization process of students [53]. As important adults in the family and school environment, parents and teachers interact with each other to affect student development [54]. From the perspective of social support theory, social support is a selective behavior that people give material and spiritual help for free to disadvantaged groups in society [55]. Social supporters include people who can have a positive meaning for suffering individuals around them, such as family members, friends, relatives, teachers, etc. In school bullying behavior of middle school students, social support can enable students to maintain positive emotional feelings and physical and mental conditions in a state of psychological stress, avoiding or reducing the harm of school bullying behavior to students [55].

Teachers are one of the main sources of social support for teenagers [56]. Teacher support refers to the behavior and attitude that students perceived for teachers’ support in their studying and life, which mainly includes cognitive support, ability support and emotional support [57]. Previous studies have shown that teachers’ emotional support is more important than cognitive support and ability support [58,59], and the teacher–student relationship have significant negative effects on students’ bullying [60,61]. When students are bullied at school, and if teachers give positive and active attention and support to the bullied students, such as severely criticizing the bullying behavior, criticizing the bully, or making the bully apologize to the bullied students, etc., and giving the victims more emotional support, it will reduce the psychological harm of bullied students and make them feel warm psychologically to a certain extent [55]; however, if when students are bullied on campus teachers choose to “pretend not to see” or ignore them, this will aggravate the feeling of helplessness and despair of the bullied student to a certain extent, which makes it difficult for them to form a sense of trust and dependence on school, and the sense of belonging to the school is correspondingly reduced [62]. Therefore, at the same level of school bullying, compared with students with higher teacher support, students with lower teacher support will find it difficult to feel a higher sense of belonging. Therefore, we propose hypothesis H3:

**H3.**  *Teacher support moderates the first half of the path that school bullying affects peer cooperation through school belonging. Compared with students with higher perceived teacher support, students who perceived lower teacher support experience school bullying will have greater negative predictive effect on their sense of school belonging.*

Similarly, based on the social ecosystem theory, “Human living environment is a complete ecosystem”, multiple systems including adolescent’s family and school are interrelated, and family experience can affect school experience. According to the social capital theory [63], as an important family social capital, family support in bullying behavior is embodied in the emotional, informational, and material help provided by parents to children after the bullying occurs. Studies have found that after their children are bullied, most parents will take their children to school to find teachers to solve the problem, and some parents will directly ask the parents of bully for an explanation [55]. These behaviors will reduce the psychological harm of the child school bullying to a certain extent. On the contrary, when the children are facing school bullying, parents choose to let the child “temporarily compromise, and find a chance to retaliate” or “compromise and give up or tell the teacher” may not be able to give the children enough emotional support, and the negative impact of children school bullying cannot be effectively mitigated. 

Meanwhile, previous studies have found that family factors also affect individual cooperative behavior. Xie et al. found that parents’ work values had a certain impact on children’s cooperation tendency [64]. The more parents attach importance to economic interests, the lower the child’s cooperative tendency; the more dominant their parents were, the less cooperative their children were. In addition, children who share good receptivity with parents are more cooperative and less aggressive or argumentative; children whose mothers neglected them and excessively restricted them showed less cooperative behavior during activities [65]. Therefore, it can be seen that parents’ support is also external protective factor that reduces the impact of the bullying injury; however, since parents’ support is not directly involved in school activities, it may not affect students’ cooperative behavior through school belonging but may directly moderate the impact of school bullying on cooperative behavior. We propose hypothesis H4:

**H4.**  *Parents’ support moderates the negative impact of school bullying on peer cooperation. Compared with students with higher parents’ support, students with lower parents’ support experience school bullying will have greater negative predictive effect on their peer cooperation.*

**Figure 1:**
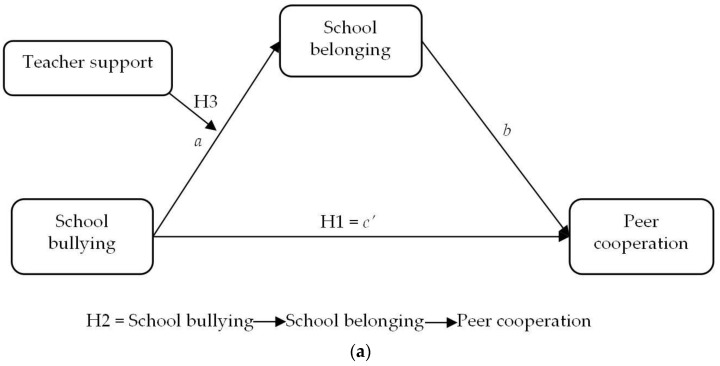

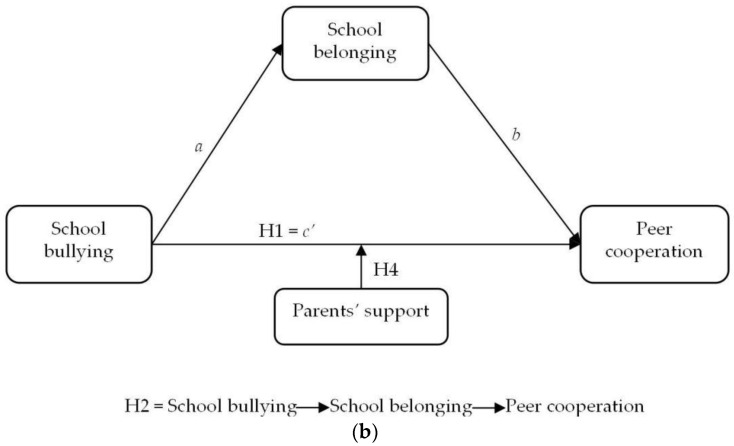
The hypothetical framework model of the study. (**a**) The moderated mediating effect of school bullying on peer cooperation (teacher support). (**b**) The effect of school bullying on peer cooperation (parents’ support).

In summary, this study conceptualizes a moderated mediation model to explore the mediating role of school belonging in the impact of school bullying on students’ peer cooperation and the moderating roles of teacher and parents’ support (see Figure 1). 

## 2. Materials and Methods

### 2.1. Materials

This study’s data were sourced from the PISA 2018 survey database, covering four provinces and cities in mainland China (Beijing City, Shanghai City, Jiangsu Province, and Zhejiang Province). The PISA test focuses on students’ academic performance in reading, math, and science, as well as their mental health and social development. All variables used in this study were derived from the PISA surveys. A detailed introduction to PISA can be found in Appendix A.

We initially downloaded the 2018 Global Student Questionnaire data file from the PISA website https://www.oecd.org/pisa/data/2018database/ (accessed on 15 October 2021), selecting the data specific to mainland China. These data encompassed 12,058 middle school students aged 15 (ranging from 15 years and 3 months to 16 years and 2 months) from 361 schools. The average class sizes varied from 18 to 53 students, with a mean of 38 students per class. After excluding samples with missing data and those that did not meet the statistical criteria, we proceeded to analyze the data. 

### 2.2. Research Variables

#### 2.2.1. Peer Cooperation

This is the outcome variable. The PISA 2018 background questionnaire asked students to rate the truthfulness of statements about peer cooperation at their school on a Likert scale from 1 (Not at all true) to 4 (Extremely true). The sum of the scores from four questions formed the peer cooperation index, ranging from 4 to 16. A higher score indicates a higher perceived level of peer cooperation. The Cronbach’s alpha coefficient for these items was 0.934, showing high internal consistency reliability.

#### 2.2.2. School Bullying

This is the predictive variable. The PISA 2018 questionnaire surveyed students’ experiences of physical, relational, and verbal bullying. Students rated the frequency of six different bullying-related experiences on a scale where 1 represented “Never or almost never” and 4 indicated “Once a week or more”. The cumulative score of these items, ranging from 6 to 24, represented the severity of bullying. According to the reliability and validity of the formative indicators [66,67], a multi-collinearity test was performed on the six items, yielding VIF values between 1.552 and 2.041, which are within the acceptable range (smaller than 3.3), indicating no multi-collinearity issues.

#### 2.2.3. School Belonging

This is the mediating variable. The PISA 2018 questionnaire measured students’ sense of school belonging through six items, rated on a Likert scale from 1 (Strongly agree) to 4 (Strongly disagree). The scores were reversed for three of the questions (question 2, 3 and 5), and the cumulative score ranged from 6 to 24. A higher score reflects a stronger sense of belonging. The Cronbach’s α coefficient for this scale was 0.832.

#### 2.2.4. Teacher Support

This is the moderating variable. This variable measured the perceived cognitive and emotional support from teachers, as rated by students in their language classes. The responses were rated on a Likert scale from 1 to 4, with the scores then reversed and summed to create an index ranging from 4 to 16. A higher score indicates greater perceived teacher support. The Cronbach’s alpha coefficient for these items was 0.864.

#### 2.2.5. Parents’ Support

This is also the moderating variable. This variable measured students’ perceived emotional support from their parents, based on three statements rated from 1 (Strongly disagree) to 4 (Strongly agree). The cumulative score ranged from 3 to 12, with higher scores indicating greater parental emotional support. The Cronbach’s alpha coefficient for these items was 0.908.

### 2.3. Statistical Analyses

Descriptive statistics, reliability analysis, and correlation analysis were performed using SPSS 24.0. The mediation model and moderated mediation model tests were conducted using Hayes’ PROCESS program [68], a computational tool available for SPSS and SAS that facilitates moderated mediation analysis. The significance of regression coefficients was tested using the Bootstrap method with a 95% confidence interval, based on 5000 repeated samplings.

### 2.4. Common Method Biases Test

The use of self-reported data collection can lead to common methodology biases. To address potential biases from self-reported data, anonymous surveys and reverse scoring of some questions were employed. Additionally, Harman’s single factor test was used to assess common method biases. The results revealed five factors with eigenvalues greater than 1, and the first factor explained only 27.699% of the variance below the 40% threshold, suggesting no significant common method biases in this study [69].

## 3. Results

### 3.1. Descriptive Statistics and Correlation Matrices for the Variables

Correlation analysis of the study variables revealed significant negative correlations between school bullying and peer cooperation, school belonging, teacher support, and parents’ support. Conversely, peer cooperation showed significant positive correlations with school belonging, teacher support, and parents’ support. Additionally, significant positive correlations were observed among the three variables of school belonging, teacher support, and parents’ support (see Table 1).

### 3.2. Moderated Mediation Model Testing

First, we tested the mediating role of school belonging in the impact of school bullying on peer cooperation using Hayes’ PROCESS procedure [68] and Wen and Ye’s guidelines [70], employing Model 4 in SPSS. The process involved 5000 bootstrap estimates for constructing 95% bias-corrected confidence intervals (CIs). 

The results indicated that school bullying significantly negatively affected students’ school belonging (*a* = −0.399, *SE* = 0.010, *t* = −38.064, *p* < 0.001, 95% *CI* = [−0.419, −0.378]), and school belonging significantly positively affected peer cooperation *(b* = 0.345, *SE* = 0.008, *t* = 43.355, *p* < 0.001, 95% *CI* = [0.330, 0.361]). The indirect effect, calculated using the Bootstrap method, was significant (*Effect* = −0.138, Boot *SE* = 0.006, and 95% *CI* = [−0.149, −0.127]). Moreover, with school bullying and school belonging in the regression equation, the direct effect of school bullying on peer cooperation was significant (*c*′ = −0.043, *SE* = 0.010, *t* = −4.546, *p* < 0.001, 95% *CI* = [−0.062, −0.025]) (see Table 2). The deviation corrected percentile Bootstrap test shows that school belonging plays a part mediating effect in the impact of school bullying on the peer cooperation (*ab* = −0.138, Boot *SE* = 0.006, 95% *CI* = [−0.149, −0.127]). Ratio of indirect to total effect is *ab*/(*ab* + *c*′) = 76.24%.

To better explain the validity of mediating effects, we calculated the mediation effect size in two ways: R-squared mediation effect size and Preacher and Kelley (2011) Kappa-squared. The results showed that while the effect size of the R-squared mediation effect was not large, the Preacher and Kelley (2011) Kappa-squared had medium effect and was acceptable, affirming the validity of our model [71]. So, we reported the effect size without overemphasizing its magnitude.

Secondly, in order to assess the moderating role of teacher support, we utilized Model 7 within the PROCESS analytical framework. Our moderated mediation analysis encompassed the estimation of three distinct regression equations: Equation (1) evaluated the total effect of school bullying on peer cooperation; Equation (2) examined the moderating influence of teacher support on the association between school bullying and school belonging; Equation (3) appraised the predictive impact of school belonging on peer cooperation, with standardization applied to all predictors. The model’s validity was confirmed by the following: (a) a significant total effect of school bullying on peer cooperation in Equation (1); (b) a notable main effect of school bullying on school belonging and a significant interaction between teacher support and school bullying in Equation (2); and (c) a significant predictive effect of school belonging on peer cooperation in Equation (3), as supported by references [68,70].

*Y* = *i_Y_* + *c′X* + *bM* + *e_Y_* (1)

*M* = *i_M_* + *a*_1_*X* + *a*_3_*XW* + *e_M_* (2)

*Y* = *i_Y_* + *bM* + *e_Y_* (3)

*Y* represents peer cooperation, *X* represents school bullying, *M* represents school belonging, and *W* represents teacher support.

Table 3 presents the results, affirming the aforementioned criteria (a), (b) and (c). The moderated mediation effect yielded an index value of −0.012 (Boot *SE* = 0.003, 95% *CI* = [−0.019, −0.006]). In line with Hayes’ study [72], this effect is statistically significant, thereby validating the model. This substantiates that the initial process through which school bullying impacts peer cooperation via school belonging is indeed moderated by teacher support. Furthermore, examining the conditional indirect effects at specific levels of teacher support revealed that at a low level of teacher support (1 *SD* below the mean), the indirect effect of school bullying on peer cooperation through school belonging was smaller (*index* = −0.108, Boot *SE* = 0.007, 95% *CI* = [−0.119, −0.097]). Conversely, at a high level of teacher support (1 *SD* above the mean), this indirect effect was more pronounced (*index* = −0.131, Boot *SE* = 0.006, 95% *CI* = [−0.145, −0.118]). Thus, the indirect effect of school bullying on peer cooperation varies in tandem with changes in the level of teacher support, intensifying as teacher support increases.

**Figure 2:**
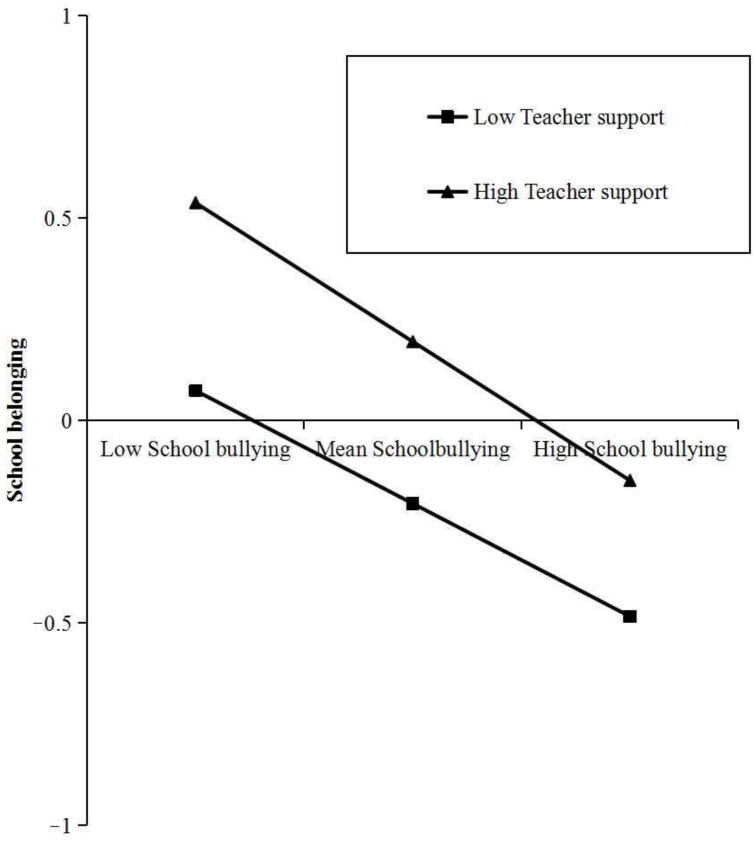
The moderating effect diagram of teacher support on the relationship between school bullying and school belonging.

In order to explain the interaction effect more clearly between school bullying and teacher support, we divided teacher support into high and low groups according to the mean plus or minus one standard deviation (*M* ± *SD*), conducted a simple slope test, and drew a simple effect analysis diagram (Figure 2). The results showed that when teacher support is high (*M* + *SD*), school bullying had significant negative prediction on school belonging (*B_simple_* = −0.339, *t* = 27.478, *p* < 0.001); when teacher support is low (*M* − *SD*), the negative prediction effect of school bullying on school belonging was weakened (*B_simple_* = −0.280, *t* = 28.442, *p* < 0.001; *B_simple_* = −0.339 decreases to *B_simple_* = −0.280).

For the moderating effect of parents’ support, Model 5 in the PROCESS procedure was used. The model required estimating three regression equations: Equation (1) for the moderating effect of parents’ support on the relationship between school bullying and peer cooperation, Equation (5) for the predictive effect of school bullying on school belonging, and Equation (6) for the predictive effect of school belonging on peer cooperation. The conditions for a significant moderating effect were: (a) Equation (4) showing a significant main effect of school bullying on peer cooperation and a significant interaction effect of parents’ support and school bullying; (b) Equation (5) demonstrating a significant predictive effect of school bullying on school belonging; (c) Equation (6) indicating a significant predictive effect of school belonging on peer cooperation.

*Y* = *i_Y_* + *c*′*X* + *c*_3′_*XW*+ *e_Y_* (4)

*M* = *i_M_* + *a*_1_*X* + *e_M_* (5)

*Y* = *i_Y_* + *bM* + *e_Y_* (6)

*Y* represents peer cooperation, *X* represents school bullying, *M* represents school belonging, and *W* represents parents’ support.

The model met these conditions, indicating that the direct process of school bullying affecting peer cooperation is moderated by parents’ support, with the index value of the moderated effect *Index* = −0.111, Boot *SE* = 0.005, 95% *CI* = [−0.121, −0.102] (see Table 4). 

**Figure 3:**
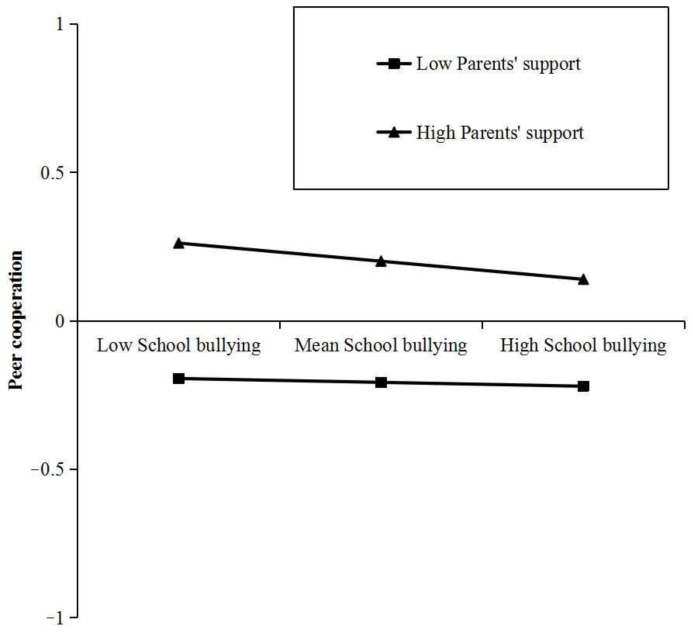
The moderating effect diagram of parents’ support on the relationship between school bullying and peer cooperation.

In order to explain the interaction effect more clearly between school bullying and parents’ support, we divided parents’ support into high and low groups according to the mean plus or minus one standard deviation (*M* ± *SD*), conducted a simple slope test, and drew a simple effect analysis diagram (Figure 3). The results showed that when parents’ support is high (*M* + *SD*), school bullying has significant negative prediction on peer cooperation (*B_simple_* = −0.062, *t* = −4.878, *p* < 0.001); when parents’ support is low (*M* − *SD*, the negative prediction effect of school bullying on peer cooperation was not significant (*B_simple_* = −0.013, *t* = −1.315, *p* < 0.189).

## 4. Discussion

This study, grounded in social cognitive theory, the frustration–aggression hypothesis, and group identity theory, reveals the relationship between school bullying and peer cooperation and its mechanisms. The key findings are twofold: firstly, the study illustrates “how school bullying works” by influencing peer cooperation through the mediating role of school belonging. Secondly, it dissects “when is more important”, showing that the initial part of this intermediary process is moderated by teacher support. Students with higher perceived teacher support experience a greater negative impact on their sense of school belonging when subjected to school bullying. Additionally, the process by which school bullying directly affects peer cooperation is moderated by parents’ support. Students with higher parents’ support experience a more significant negative impact on peer cooperation when subjected to school bullying. These findings have substantial theoretical significance and practical value for the scientific prevention and intervention of school bullying.

### 4.1. The Direct Effect of School Bullying on Peer Cooperation

This study substantiates that school bullying significantly and negatively predicts students’ peer cooperation, aligning with the principles of the frustration–aggression hypothesis and group identity theory. This diminished perception of peer cooperation may stem from students exhibiting aggressive or apathetic behaviors as a response to the frustrations experienced due to bullying [29]. As victims of school bullying often do not perceive themselves as belonging to the same social group as their aggressors, this group heterogeneity hinders cooperative efforts [50]. Peer relationships are pivotal in adolescent development, with adolescents spending a considerable portion of their time engaged in academic and extracurricular activities with peers. Positive peer interactions foster personality development and maturity. However, bullied students may find themselves alienated from these interactions and less inclined to participate in school activities, adversely affecting classroom participation rates, enrollment, and academic performance [73]. Recent studies corroborate these findings, indicating that school bullying diminishes students’ inclination towards cooperation [42]. This study echoes these findings, underscoring the substantial negative impact of school bullying on students’ perceptions of and engagement in interpersonal cooperation. Additionally, the experience of school bullying can precipitate severe negative mental health outcomes, such as increased suicidal ideation and attempts [74,75], anxiety disorders [76], psychiatric symptoms [77], depression [78], and sleep disturbances [79]. Thus, the issue of school bullying warrants concerted attention from all societal sectors.

### 4.2. The Mediating Role of School Belonging

Upon establishing the direct effect of school bullying on peer cooperation, this study further identifies school belonging as a mediating factor in this relationship. Specifically, school bullying undermines peer cooperation by eroding students’ sense of belonging within the school environment. According to Maslow’s hierarchy of needs, the desire for group belonging is fundamental during adolescence. School belonging encompasses students’ ideological, emotional, and psychological identification with, and active participation in, their educational institution, hoping for acceptance by their peers. Given that schools are primary socialization environments during this critical period of value formation, the impact of bullying on students’ sense of belonging is profound [80,81]. Teenagers are in a critical period of forming correct values about the world, life, and themselves. They value the acceptance, care, and identification of others. If they are bullied at school, it is very difficult for them to form a school belonging [40]. 

Students who were bullied at school would have suffered physical and psychological trauma in school life. They do not feel the collective acceptance and recognition of themselves. Then, it is very difficult to accredit school and the group they belong to from the ideological and emotional aspects, and to have a sense of school belonging. They cannot feel their value in interacting with others, and do not feel the emotion of being a whole with others. Naturally, the degree of perception of peer cooperation will also be reduced.

### 4.3. The Moderating Role of Teacher Support and Parents’ Support

Beyond the mediating role of school belonging, this study also unveils the moderating effects of teacher and parents’ support in the relationship between school bullying and peer cooperation. Specifically, teacher support moderates the first half of the indirect pathway (“school bullying–school belonging–peer cooperation”), while parents’ support moderates the direct effect of school bullying on peer cooperation. Intriguingly, in contrary to hypotheses H3 and H4 and diverging from previous research and theories, the study reveals that lower levels of teacher and parents’ support are associated with a diminished negative impact of school bullying on school belonging and peer cooperation, respectively.

This finding challenges traditional views in social support theory [82,83], which posit that higher levels of perceived social support typically confer positive emotional energy and effective coping mechanisms, thereby buffering negative impacts [84]. Also, according to the Mills’ theory of important others, parents, teachers, and peers are important others in the process of socialization of middle school students. Teacher support and parents support are important protective factors [85]. Teacher support is an important manifestation of teachers’ listening, encouragement, and respect for students [86]. Students are more likely to seek help from teachers when they encountered a problem or in a difficult situation [40]. Parents’ support is also a protective factor from the impact of school bullying [85]. Studies have found that undesirable parent–child communication can lead to students’ anti-social behaviors such as aggression and hostility [87]. A higher level of family support is associated with a lower risk of bullying [88]. As the social capital of the family, parents are one of the important subjects in the prevention and treatment of school bullying, and high-quality parental emotional participation will reduce the frequency of school bullying [89]. 

However, our findings offer a different perspective on social support, of which excessive support, particularly in the context of overprotective or indulgent parenting, may inadvertently render students more vulnerable to the detrimental impacts of bullying. According to our results, when the level of teacher support and parents’ support were lower, the negative impact of school bullying were smaller. We think that it may be caused by the following reasons. In general, Chinese parents and teachers give great support to young student’s life and study, but sometimes it may exceed a certain limit. Over-protection and even spoiling of their children has been observed, especially by Chinese parents who often do everything for their children and make every effort to protect them from the outside injury. These over-protected children are like “flowers in a greenhouse” [90]. This “greenhouse effect” posits that overly shielded students, accustomed to having problems resolved for them, may experience more pronounced negative effects when exposed to bullying. However, for students who lack social support, they are accustomed to less support may develop resilience, lessening the impact of bullying on their sense of cooperation. So, it looks as if social support also has a certain degree of impact, and if excessive, it may cause the indulgence of the individual, but does not play a protective role. These insights suggest a nuanced understanding of social support, emphasizing the need for balance to avoid fostering dependency and vulnerability.

### 4.4. Suggestions on the Results

In the process of children’s growth, cooperation is a fundamental mode of social interaction and learning. The development of cooperative skills and abilities is essential for successful social and group integration [65]. Studies from the social aspect of children prove that cooperation and friendliness are positively correlated with prosocial behavior and peer acceptance, while aggression and destructive behavior lead to peer rejection [91]. Given the increasing prevalence and societal concern regarding school bullying, a multi-faceted approach involving government, parents, teachers, schools, and society at large is imperative for its prevention and treatment. 

While maintaining a balanced approach, we believe that parents’ support and teacher support are still very important factors. Both family and school environments are pivotal in combating school bullying, with home–school collaboration enhancing anti-bullying efforts. Parents should offer more psychological and emotional support, especially to students facing academic challenges, fostering confidence and resilience rather than resorting to criticism. Based on our previous research, certain student demographics, such as boys, students with repeated grades, truancy, and tardiness in the week before the test, and students with lower ESCS (economic, social, and cultural status) were more likely to experience more school bullying [92]. Therefore, teachers should pay more attention to these students groups, think about the causes of bullying behavior from a variety of perspectives, and use multiple ways to deal with bullying behavior to help students learn correct attitudes and behaviors. In addition, teachers must pay more attention to the circle of friends and interaction between students, and if a particular student is excluded, even in an isolated situation, once it is found that there are signs of bullying behavior among students, they should provide appropriate treatment at the first time to prevent the occurrence of bullying. 

### 4.5. Limitations and Future Research Directions

This study, while comprehensive, is constrained by its reliance on the PISA test database, potentially omitting other relevant factors influencing students’ experiences of school bullying and peer cooperation. Future research should consider employing alternative databases or custom-designed questionnaires for a more exhaustive analysis.

Additionally, while the cross-sectional design of this study is theoretically grounded, it still cannot fully infer the causal relationship between school bullying and peer cooperation. Longitudinal studies are recommended to further elucidate these relationships..

Finally, we treat school bullying as a continuous variable in this study. Future research could employ latent profile analysis (LPA) to identify potential subgroups of people being bullied, non-bullied, or popular students and conduct comparative studies. By comparatively analyzing individuals’ physical characteristics (such as obesity, disability), social characteristics (such as race), psychological characteristics (such as introversion), or other aspects, we ultimately hope to uncover the root causes of vulnerability to bullying and develop preemptive strategies.

## 5. Conclusions

Utilizing data from the PISA 2018 survey, this study delves into the impact of school bullying on 15-year-old students’ peer cooperation and its mediating and moderating mechanisms. The key findings are as follows.

First, school bullying had a significant negative predictive effect on students’ peer cooperation, that is, greater bullying severity correlates with lower levels of peer cooperation. Secondly, school belonging partially mediates the relationship between school bullying and peer cooperation, indicating that bullying adversely affects peer cooperation by diminishing students’ sense of school belonging. Finally, the study identifies significant moderating effects of teacher support on the indirect effect of school bullying on peer cooperation, and of parents’ support on the direct effect of bullying on peer cooperation. Notably, decreased levels of teacher and parents’ support were found to mitigate the negative impacts of school bullying, providing a novel perspective on the role of social support in the context of school bullying.

## Figures and Tables

**Table 1:** Descriptive statistics and correlation coefficient matrix of each variable.

Variables	*M*	*SD*	1	2	3	4	5
1. Peer cooperation	11.388	2.767	1				
2. School bullying	7.604	2.931	−0.171 **	1			
3. School belonging	17.716	3.297	0.404 **	−0.333 **	1		
4. Teacher support	13.576	2.769	0.286 **	−0.175 **	0.246 **	1	
5. Parents’ support	9.990	1.929	0.304 **	−0.144 **	0.286 **	0.202 **	1

Note: *M* is the mean and *SD* is the standard deviation. ** *p* < 0.01.

**Table 2:** Conditional indirect effects of school bullying on peer cooperation through school belonging.

Outcome Variables	Predictive Variables	Effect	*SE*	*t*	*p*	*LLCI*	*ULCI*
**Total effect**							
Peer cooperation	(constant)	12.767	0.078	162.843	<0.001	12.614	12.921
	School bullying	−0.181	0.010	−18.660	<0.001	−0.200	−0.162
**Direct effect**							
	(constant)	5.603	0.181	31.027	<0.001	5.249	5.957
Peer cooperation	School bullying	−0.043	0.010	−4.546	<0.001	−0.062	−0.025
**Indirect effect**							
School belonging	(constant)	20.752	0.085	245.205	<0.001	20.586	20.918
	School bullying	−0.399	0.010	−38.064	<0.001	−0.419	−0.378
Peer cooperation	(constant)	5.603	0.181	31.027	<0.001	5.249	5.957
	School belonging	0.345	0.008	43.355	<0.001	0.330	0.361
		**Effect**	**Boot *SE***	***t***	***p***	**Boot *LLCI***	**Boot *ULCI***
Peer cooperation	School bullying	−0.138	0.006	/	/	−0.149	−0.127
Ratio of indirect to total effect of School bullying on Peer cooperation							
School belonging		0.760	0.051	/	/	0.673	0.876
R-squared mediation effect size (R-sq_med)							
School belonging		0.028	0.003	/	/	0.022	0.033
Preacher and Kelley (2011) Kappa-squared							
School belonging		0.128	0.005	/	/	0.118	0.138

Note: Bootstrapping based on n = 5000 subsamples. *CI* indicates confidence interval; *LL* = lower limit, indicating the lower confidence interval; *UL* = upper limit, indicating upper confidence interval. Bootstrap SE indicates the standard error after Bootstrap is executed.

**Table 3:** Results of the moderated mediating effect test of teacher support.

Outcome Variables	Predictive Variables	*R* ^2^	*F*	*β*	*SE*	*t*	*p*	*LLCI*	*ULCI*
School belonging	(constant)	0.149	677.969	−0.005	0.009	−0.593	0.553	−0.022	0.012
	School bullying			−0.311	0.009	−34.143	<0.001	−0.329	−0.293
	Teacher support			0.200	0.009	22.782	<0.001	0.183	0.217
	School bullying × Teacher support			−0.032	0.007	−4.508	<0.001	−0.046	−0.018
Peer cooperation	(constant)	0.164	1138.785	<0.001	0.008	0.031	0.976	−0.016	0.017
	School bullying			−0.041	0.009	−4.586	<0.001	−0.059	−0.024
	School belonging			0.388	0.009	43.270	<0.001	0.370	0.406
Conditional indirect effect at specific levels of the moderator									
**Moderator: level of Teacher support**				***β***	**Boot *SE***	***t***	***p***	**Boot *LLCI***	**Boot *ULCI***
*M* − *SD*				−0.108	0.006	/	/	−0.119	−0.097
Mean				−0.121	0.005	/	/	−0.131	−0.110
*M* + *SD*				−0.131	0.007	/	/	−0.145	−0.118
Index of moderated mediation									
				***Index***	**Boot** ***SE***	***t***	***p***	**Boot *LLCI***	**Boot *ULCI***
	Teacher support			−0.012	0.003	/	/	−0.019	−0.006

**Table 4:** Results of the moderated effect test of parents’ support.

Outcome Variables	Predictive Variables	*R* ^2^	*F*	*β*	*SE*	*t*	*p*	*LLCI*	*ULCI*
School belonging	(constant)	0.110	1442.695	<0.001	0.009	0.051	0.960	−0.017	0.018
	School bullying			−0.334	0.009	−37.983	<0.001	−0.352	−0.317
Peer cooperation	(constant)	0.201	732.234	−0.003	0.008	−0.394	0.693	−0.02	0.013
	School bullying			−0.037	0.009	−4.117	<0.001	−0.055	−0.02
	School belonging			0.333	0.009	36.661	<0.001	0.315	0.35
	Parents’ support			0.204	0.009	23.489	<0.001	0.187	0.221
	School bullying × Parents’ support			−0.024	0.007	−3.525	<0.001	−0.038	−0.011
Indirect effect of School bullying on Peer cooperation									
				***Index***	**Boot *SE***	***t***	***p***	**Boot *LLCI***	**Boot *ULCI***
	School belonging			−0.111	0.005	/	/	−0.121	−0.102

## Data Availability

Publicly available datasets were analyzed in this study. This data can be found here: https://www.oecd.org/pisa/data/2018database/ (accessed on 15 October 2021).

## Appendix A

### Appendix A.1. Overview of PISA and Its Questionnaires

This study’s data were sourced from the 2018 PISA survey database. Conducted by the Organization for Economic Cooperation and Development (OECD), PISA assesses 15-year-old students worldwide in reading, mathematics, and science. The objective is to determine their readiness to engage in modern societal and economic activities. The assessment evaluates not just knowledge replication but also the application of learned concepts to new situations, both inside and outside of school. Initiated in 2000, PISA is conducted every three years, with detailed assessments in the three core subjects rotating every nine years. Along with these assessments, PISA includes background questionnaires that gather data on students’ family backgrounds, school environments, attitudes, beliefs, and experiences. Recent iterations have included surveys on contemporary issues, such as bullying (introduced in 2015) and perceived peer cooperation (added in 2018). PISA also involves parents, teachers, and school principals or leaders, with the latter providing insights into school management and learning environments. Since 2000, PISA has involved over 90 countries and over 3 million students, offering extensive global data on student education.

### Appendix A.2. Data Collection Methodology

The 2018 PISA data encompass information from 75 countries and economies, targeting 15-year-olds in grade 7 and above. The sampling method is a two-stage stratified design. In the first stage, schools with 15-year-old students are systematically chosen from a national list based on the School Sampling Framework. This Probability Proportional to Size (PPS) sampling sorts schools into distinct groups based on specific characteristics to enhance sample estimate accuracy. In the second stage, students from these selected schools are sampled. Each participating country or economy in the Computer-based Assessment (CBA) and Global Competitiveness (GC) aims for a target cluster size (TCS) of 42 students. Countries or economies participating only in the Paper Assessment (PBA) or in the CBA without GC aim for a TCS of 35. If a school’s list of 15-year-olds is shorter than the target number, all students on the list are included.
